# Burden of Prostate Cancer in China, 1990–2019: Findings From the 2019 Global Burden of Disease Study

**DOI:** 10.3389/fendo.2022.853623

**Published:** 2022-05-25

**Authors:** Fuquan Wang, Chenchen Wang, Haifa Xia, Yun Lin, Dingyu Zhang, Peng Yin, Shanglong Yao

**Affiliations:** ^1^ Department of Anesthesiology, Union Hospital, Tongji Medical College, Huazhong University of Science and Technology, Wuhan, China; ^2^ Institute of Anesthesia and Critical Care Medicine, Union Hospital, Tongji Medical College, Huazhong University of Science and Technology, Wuhan, China; ^3^ Jinyintan Hospital, Tongji Medical College, Huazhong University of Science and Technology, Wuhan, China; ^4^ National Center for Chronic and Noncommunicable Disease Control and Prevention, Chinese Center for Disease Control and Prevention, Beijing, China

**Keywords:** burden of disease, disability-adjusted life-years, public health, epidemiology, prostate cancer

## Abstract

Our study is the first to illustrate the age and geographic distribution differences in the epidemiology of prostate cancer from 1990 to 2019 in China. Prostate cancer (PC) is a malignant tumor derived from prostate epithelial cells and is one of the most commonly diagnosed cancers in men. In recent years, the global incidence and the annual deaths number of PC showed a continuous increase, which has caused a huge disease burden on human health. In terms of the global average, the incidence and mortality of PC in China are relatively low. However, the age-standardized incidence rate of PC was 17.3/100,000 in 2019 in China, with a 95.2% rise compared to 1990, while the global growth rate of incidence rate over the same period is 13.2%. This showed that the development trend of PC in China is not optimistic. There are few precise studies on the epidemiology of PC in China. After the general analysis strategy used in the Global Burden of Diseases, Injuries and Risk Factors Study (GBD) 2019, we elaborated on the incidence, mortality, and disability-adjusted life-years (DALYs) and the corresponding age-standardized rate of the Chinese PC population from 1990 to 2019 according to different ages and provinces. We used joinpoint regression analysis to estimate the incidence and mortality trends. Our analysis shows that elderly people over 80 are still the main focus of incidence and death. The epidemiology and disease burden of PC of different provinces in China show obvious regional differences, and some certain provinces such as HongKong, Macao, and Zhejiang should be paid more attention. More targeted and effective strategies should be developed to reduce the burden of PC in China.

## Introduction

Prostate cancer (PC) ranks second in the current global incidence of male malignant tumors and is the fifth leading cause of cancer deaths in male patients (1). In 2019, there were an estimated 1410451 new cases of PC worldwide, and about 486836 patients died of PC. The age-standardized incidence rate and age-standardized death rate were 38.6/100,000 and 15.3/100,000. In terms of the global average (2), the incidence of PA in China is still at a relatively low level, but it has shown a significant upward trend in recent years. With the increasing aging of the Chinese population, the continuous westernization of diet and lifestyle, etc., the development of PA in China is even less optimistic (3–5).

Considering the major threat that PC poses to human health, there is an urgent need to accurately assess the epidemiological trends of PC. PC is particularly common in developed countries (6), so previous epidemiological studies mainly focus on the PC disease burden among different countries (2, 7), and more concentrated on European and American countries (8, 9). Currently, few targeted studies are focusing on the burden of PC in China. Due to the differences in important factors such as economic development level and medical level, the incidence, and death of PC have large spatial distribution differences (10, 11). China is made up of many provinces and there are varying degrees of differences in the economy, medical level, lifestyle, and the degree of population aging among different provinces. Therefore, there are bound to be differences in the epidemiological characteristics of PC among these provinces. Regrettably, there is no comparison of the epidemiological characteristics and disease burden of PC among different provinces in China. Our research is beneficial to the adjustments to the medical strategy by governments and medical institutions according to the development level and regional characteristics of PC. Clarifying the epidemiological distribution and trend of the incidence and death of PC in different provinces of China can make public health resources more reasonable distribution so that PC patients can obtain more balanced medical resources, which is beneficial for them to receive better treatment.

Based on the epidemiological data related to PC from the Global Burden of Diseases, Injuries and Risk Factors Study (GBD) 2019, our research clarified the current status, the spatial patterns, and temporal trends of PC in China according to age and region. Our research can help the public and policymakers assess the current PC disease burden, coordinate resource allocation, and improve the efficiency of PC prevention and treatment.

## Materials and Methods

### Data Sources

The GBD 2019 includes more than 3.5 billion estimates of 286 causes of death, 369 diseases and injuries, and 87 risk factors in 204 countries and regions (12, 13). Input data were extracted from various sources were extracted from censuses, including household surveys, civil registration and vital statistics, disease registries, health service use, air pollution monitors, and so on. It is the largest and most comprehensive quantification of health loss across regions and times research. We obtained the related data on PC from the Global Health Data Exchange (GHDX, http://ghdx.healthdata.org/gbd-results-tool). Data of China were mainly obtained from the following sources: the Maternal and Child Surveillance System, the Chinese Center for Disease Control and Prevention cause-of-death reporting system, cancer registries, the Disease Surveillance Point system, and reports from Hong Kong and Macao (14). The data included the annual case data and the age-standardized rates of the incidence, the deaths, the years of life lost (YLLs), the years of life lived with disability (YLDs), the disability-adjusted life-years (DALYs) of PC in 204 countries, and regions around the world from 1990 to 2019. International Classification of Diseases (ICD),10th revision codes C61‐C61.9, D29.1, and D40.0 were used to represent PC.

### Estimates of Disease Burden

GBD 2019 provides a standardized approach for estimating the prevalence, incidence, mortality, YLDS, YLLS, and DALYs of each disease (13). The general approach of GBD2019 to estimate causes of death and incidence is the same as GBD2017 (15, 16). In short, it is to first analyze data from different sources to generate a specific cause of mortality (17), then use the linear-step mixed-effects model, sociodemographic index, and general spatiotemporal Gaussian process regression to analyze the incidence and mortality data from multiple sources to obtain mortality-to-incidence ratios (MIRs) (18). The final estimated incidence of PC is obtained by dividing the estimated mortality by MIRs. YLDs are estimated as the product of the disability weight in the Bayesian regression model and the prevalence. YLLs were estimated by multiplying the estimated number of deaths with the life loss value for the corresponding age. Disability-adjusted life years (DALYs) were generated by summing of the YLLs and the YLDs.

As in the previous studies (19), we use Joinpoint regression analysis to assess the trend in the disease burden of PC by Joinpoint software (4.5.0.1). Annual percentage change (APC) and 95% confidence interval (CI) were calculated.

## Results

### Overall Findings

**Table 1** showed the overall age-standardized rate, number, and percentage change of PC from 1990 to 2019 at the global and China levels, respectively. In 1990, the incidence numbers of PC were 26,440 (20103-31,916.887) in China, and the age-standardized incidence rate was 8.9 (7.1-10.9) per 100,000. In 2019, the values of the above two indicators are 153,448 (118,400-204,943) and 17.3 (13.6-22.7) per 100,000. The death numbers and the DALYs number of PC in China in 1990 were 20,382 (15,817-24,679) and 403,112 (606,178-488,113), respectively. By 2019, it has grown to 54,391 (42,904-71,307) and 1,002,594 (794,010-1,322,635) respectively. The age-standardized DALYs rate has dropped from 125.9 (99.5-151.8) per 100,000 in 1990 to 118.9 (95.1-154.1) per 100,000 in 2019. The global average of the indicators mentioned above is higher than that of China. From 1990 to 2019, the growth rate of China’s PC incidence rate and the decline rate of DALYs rate were higher than the world level, and the decline rate of deaths rate was lower than the world average.

**Table 1:** The age-standardized rate, numbers and percent change for PC globally and for China, 1990–2019.

Variables	Global			China		
	1990	2019	Change(%)	1990	2019	Change(%)
Incidence Rate	34.1(26.8-39.7)	38.6 (33.6-49.8)	13.2	8.9(7.1-10.9)	17.3 (13.6-22.7)	95.2
Incidence Numbers	524,110 (409,133-613,005)	1,410,452 (1,227,900-1,825,766)	169.1	26,440 (20,103-31,917)	153,448 (118,400-204,943)	480.4
Deaths Rate	18.1(14.7-21.2)	15.3 (13.0-18.6)	-15.7	8.2 (6.6-10.3)	7.8 (6.2-9.9)	-5.3
Deaths Numbers	232,999 (191,398-268,882)	486,836(420,498-593,689)	108.9	20,382 (15,817-24,679)	54,391 (42,904-71,307)	166.9
DALYs Rate	286.3 (232.8-326.2)	244.1 (211.8-297.7)	-0.1	125.9 (99.5-151.8)	118.9 (95.1-154.1)	-5.6
DALYs Numbers	4,360,506(3,528,030-4,951,007)	8,644,870 (7,548,020-10,559,866)	98.3	403,112 (606,178-488,113)	1,002,595 (794,010-1,322,635)	148.7

### Temporal Trends of Burden of Prostate Cancer From 1990 to 2019

**Figure 1:**
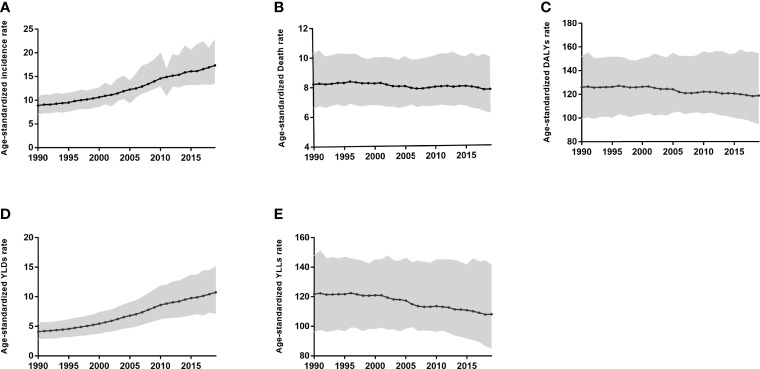
Age-standardized incidence **(A)**, Death **(B)**, DALYs **(C)**, YLDs **(D)**, and YLLs **(E)** rate for Prostate Cancer in China from 1990 to 2019.

**Figure 1** shows the related epidemiological indicators of the age-standardized rate of PC in China from 1990 to 2019. **Table 2** showed the APC of the age-standardized rate. The age-standardized incidence rate of PC increased from 1990-1997 in China (**Figure 1A**), during this period, the APC during 1990-1994 was 1.3 (0.7,1.8), 1994 to 2001 was 2.1 (1.9,2.4), 2001 to 2007 was 2.9 (2.5,3.3), 2007 to 2010 was 4.2 (2.5,6.0) and 2010 to 2019 was 1.9 (1.7,2.0), respectively.

**Table 2:** Trends in age-standardized incidence rate, death rate and DALYs rate for China, 1990–2019.

Measure	Trend	Years	Annual Percentage Change (95% CI)
DALYs rate	1	1990-2001	0.0 (-0.1,0.1)
	2	2001-2008	-0.6* (-0.8,-0.4)
	3	2008-2011	0.3 (-0.8,1.5)
	4	2011-2019	-0.4* (-0.5,-0.2)
Deaths Rate	1	1990-1997	0.3* (0.1,0.5)
	2	1997-2007	-0.6* (-0.7,-0.4)
	3	2007-2015	0.2 (-0.0,0.4)
	4	2015-2019	-0.9* (-1.3,-0.4)
Incidence rate	1	1990-1994	1.3* (0.7,1.8)
	2	1994-2001	2.1* (1.9,2.4)
	3	2001-2007	2.9* (2.5,3.3)
	4	2007-2010	4.2* (2.5,6.0)
	5	2010-2019	1.9* (1.7,2.0)

*p-value < 0.05.

**Figure 2:**
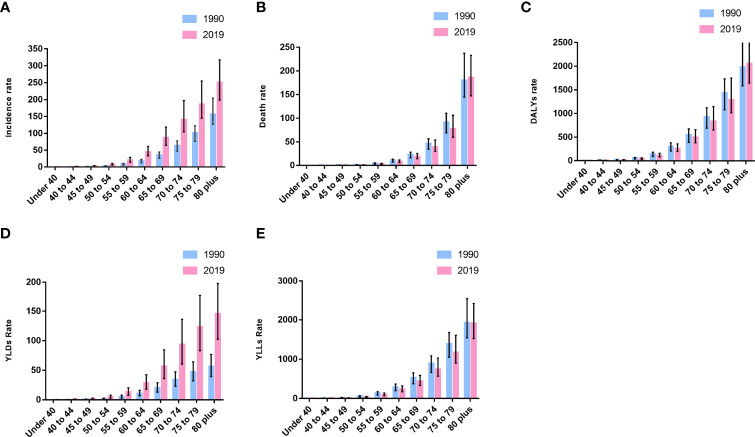
Incidence **(A)**, death **(B)**, DALYs **(C)**, YLDs **(D)**, and YLLs **(E)** rate by age for prostate cancer in China in 2019.

The age-standardized death rate and the age-standardized DALYs rate from 1990 to 2019 of PC in China showed an overall downward trend (**Figures 1B, C**). The two important parts of the DALYs rate, YLDs, and YLLs, show opposite trends (**Figure 2D, E**). The age-standardized YLLs rate gradually decreases, while the age-standardized YLDs rate increases year by year.

### Age Distribution Pattern

We compared the incidence numbers and the deaths numbers of PC patients in different age groups in China in 1990 and 2019 (**Table 3**). We found that the incidence numbers of PC in all age bands in 2019 were higher than the corresponding age range in 1990. In 2019, the incidence numbers of PC is mainly concentrated in the age bands above 60 years old, and the age group with the largest number of cases is 80 plus.

**Table 3:** The incidence and death numbers for Prostate Cancer by age bands a in 1990 and 2019.

Age Bands	1990		2019	
	Incidence numbers	Death numbers	Incidence numbers	Death numbers
Under 40	354 (248-433)	160 (113-194)	1,068 (843-1,424)	141 (112-189)
40 to 44	149 (105-188)	79 (55-98)	599 (449-830)	93 (69-126)
45 to 49	283 (186-362)	144 (97-184)	1,702 (1,227-2,435)	257 (188-361)
50 to 54	743 (493-950)	371 (247-480)	4,624 (3,329-6,638)	677 (483-973)
55 to 59	1,885 (1,257-2,458)	967 (639-1,241)	9,482 (6,747-13,565)	1,476 (1,075-2,077)
60 to 64	3,300 (2,335-4,147)	1,910 (1,311-2,407)	17,569 (13,007-24,057)	3,301 (2,459-4,542)
65 to 69	4,777 (3,468-5,974)	3,036 (2,158-3,773)	29,882 (22,282-40,959)	6,515 (4,926-8,730)
70 to 74	5,542 (4,086-6,763)	4,073 (3,026-4,970)	32,692 (24,396-45,960)	9,066 (6,944-12,600)
75 to 79	4,957 (3,739-6,006)	4,494 (3,392-5,435)	26,301 (20,423-36,011)	10,952 (8,443-15,066)
80 plus	4,450 (3,619-5,834)	5,149 (4,142-6,781)	29,528 (23,376-37,374)	21,912 (17,360-27,453)

As for the number of deaths of PC, although the total number of deaths from PC in 2019 was significantly higher than that in 1990, the number of deaths from PC in the age group under 40 in 2019 was lower than in 1990. In 2019, the death numbers in each age group increased with age, reaching a peak in the 80 plus age group. In 1990, the deaths numbers in each age group showed the same trend as in 2019 and peaked in the 80 plus age group.

The incidence, death, and DALYs rate of PC in 1990 and 2019 were compared by age groups (**Figures 2A**–**C**). In 2019, the incidence rate of each age group was significantly higher than that in 1990, but the 80 plus group of death and DALYs rate showed a difference from other groups. The PC death and DALYs rate of people under 80 years old in 2019 showed a downward trend compared with 1990, but the above two indicators of people over 80 years old are opposite.

Both YLLs and YLDs rates increase with age, and the 80plus group has the highest YLDs and YLLs rate (**Figures 2D, E**). However, the comparison of the YLDs and YLLs rates in 2019 and 1990 shows that the YLLs rate of each age group in 2019 is lower than in 1990, while the YLDs rate is just the opposite.

### Geographic Differences in China

**Figure 3:**
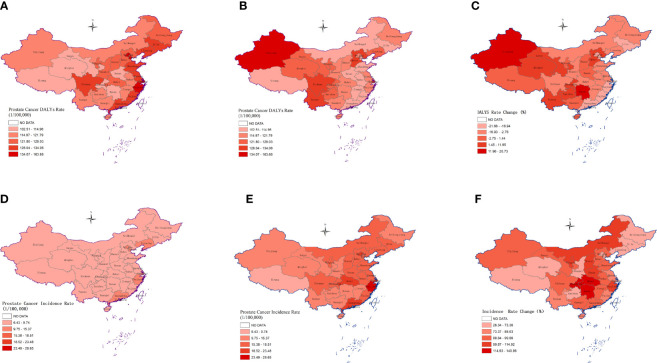
The age-standardized incidence rate of Prostate Cancer in 33 provinces of China in 1990 **(A)**, 2019 **(B)**, and the percentage change of incidence **(C)**. The age-standardized DALYs rate of Prostate Cancer in 33 provinces of China in 1990 **(D)**, 2019 **(E)** and the percentage change of incidence **(F)**.

From 1990 to 2019, the incidence of PC in different provinces of China has undergone significant changes (**Figures 3A**–**C**). In 2019, the top three provinces with age-standardized incidence rate per 100,000 were Hong Kong 28.7 (19.6,39.8), Macao 25.2 (17.1,36.0) and Zhejiang 25.0 (17.4,36.8). The age-standardized incidence rate of Xizang was 8.1 (5.6,11.1) per 100,000 in 2019, which was the lowest among the provinces. By 2019, the age-standardized incidence rate of PC in 23 provinces in China exceeded 15. In 1990, only Hong Kong had an age-standardized incidence rate of PC exceeding 15.

In 2019, the top three provinces with the highest DALYs rate of PC in China are Xinjiang 145.2 (101.5,192.2), Shanghai 130.6 (80.5,179.6), Yunnan 130.2 (102.1,173.2) (**Figure 3E**). In 1990, the three provinces with the highest DALYs rate were Shanghai 163.7 (112.7,206.2), Tianjin 146.2 (113.6,188.3), and Zhejiang 142.7 (98.1,183.5) (**Figure 3D**). From 1990 to 2019, the highest growth rate of PC disease burden was Xinjiang 20.7%, and the lowest was Tianjin -21.6% (**Figure 3F**).

## Discussion

In 1990, the incidence numbers of PC in China accounted for 5.0% of the incidence numbers of PC in the world. By 2019, the incidence of PC in China has accounted for 10.8% of the total number of incidences of PC in the world. The proportion of China PC death numbers in the world is also increasing, respectively 8.8% in 1990 and 11.2% in 2019. These indicated that Chinese PC accounts for an increasing proportion of PC patients in the world, and we need to pay more attention to the epidemiology of PC in China.

Our research found that from 1990 to 2019, one of the most obvious epidemiological features of PC in China was the continuous increase of the incidence. Moreover, the rate of increase in the incidence of PC in China is much higher than the world average. The year-on-year increase in the incidence of PC in China may be mainly due to the continuous progress of the aging of the population, the westernization of the diet and lifestyle, the active development of PSA screening, and so on.

PC is a typical senile disease, and age is one of the definite risk factors (20). The aging population in China is a particularly noteworthy issue. The proportion of the population aged 65 and over in China has increased from 5.6% in 1990 to 12.0% in 2020, and this proportion is still rising. It is estimated that the proportion of the population aged 65 and over will reach 23.7% by 2040 (21). Our research found the incidence, the mortality and the DALYs rate of PC are positively correlated with age in China. The elderly not only have a high incidence of PC but also have a high degree of malignancy and a low survival rate (22). This may be related to the weakening of body functions such as the function of important organs and immunity due to aging. The elderly over 65 should still be the focus of attention.

It is worth noting that although the incidence numbers of PC population under the age of 40 have increased, the death numbers have decreased. This phenomenon reflects the progress of medical standards in China, but it also implies that the overall age of onset of PC in China is moving forward, and it is necessary to further explore PC prevention and treatment strategies suitable for national conditions. More attention should be paid to the status of PC among young people.

Our analysis showed that the incidence of PC increased significantly from 2007 to 2010 (APC=4.21), which may be related to the 2007 Chinese Urology Guidelines that added recommendations for annual PSA testing for men over 55 years of age (23). Before 2007, there was no standard guideline for PSA testing in China. PSA testing is provided based on the experience of clinicians. The early detection and treatment of PC are of great significance to improve the duration and quality of life of the PC patients. However, PSA is not a specific indicator of PC, so PSA is still controversial in the early screening of PC.

The China Statistical Yearbook showed that Chinese residents’ consumption expenditures on meat and dairy products are increasing rapidly, and residents’ nutritional diets consume excessive amounts of protein, fat, and sugar. Chinese population’s malnutrition is becoming more and more serious. Studies have shown that increased dietary intake of animal fat, meat, and dairy products may increase the risk of PC (24–26). Unhealthy eating habits have continued to increase the rate of overweight and obesity in China. The obesity prevalence rose from 3.1% in 2004 to 8·1% in 2018 (27). Obesity is positively correlated with the incidence and mortality of high-grade advanced PC (28). Therefore, the construction of a reasonable dietary structure is of great significance in reducing the incidence of PC.

One of the exciting news is that from 1990 to 2019, the age-standardized death rate and DALYs rate showed a relatively steady decline. The main components of DALYs, YLDs, and YLLs show opposite time trends, YLLs increase year by year while YLDs decrease. It shows that with the improvement of medical standards, the overall disease burden caused by PC is on a downward trend. YLDs account for an increasing proportion of the disease burden of PC.

Previous studies have shown that there are large differences in the incidence of PC between different geographic regions (29). There are many reasons for the regional differences in the epidemiology of PC. For example, the incidence of PC in Asian populations in European and American developed countries is quite different from other ethnic groups (11), indicating that genetic background plays a certain role in the occurrence and development of PC in different ethnic groups. In addition to race and family history, which are two relatively clear risk factors for PC (30–32), diet and lifestyle among different regions may also play an important role in it. Our research showed that there are significant differences in the spatial distribution and temporal changes of PC among different provinces in China. China has a vast territory with 34 provincial-level administrative regions. There are obvious differences in environment, diet, climate, economic development level, and medical level among different regions. The severity of population aging in different provinces is different, which may be another important reason for the difference in the burden of PC disease in different provinces. In 2018, six provinces including Liaoning, Shanghai, Shandong, Sichuan, Jiangsu and Chongqing all crossed the 14.0% deep aging standard.

With the global PC incidence rate increasing year by year, the incidence of PC in China is increasing rapidly, especially in the western and central provinces of China. China has entered a society with an aging population, and the disease burden of PC will further increase. Therefore, PC should be paid more attention to cancer prevention and control in the future.

A certain degree of limitation exists in our research. On the one hand, our data is mainly based on GBD 2019, but the data may not fully represent the real situation in different provinces in China, which may lead to inaccurate estimates of disease burden. On the other hand, our research only uses age as a grouping factor and has not conducted an in-depth exploration of many factors that are closely related to the evolution of the disease burden of PC, such as the lifestyles of people in different regions.

## Conclusion

In summary, our studies suggested that PC has caused a huge health burden on the Chinese population and has obvious geographical differences. More effective strategies and policies are urgently needed to reduce the disease burden of PC and improve the quality of life of people, especially older men.

## Data Availability Statement

The original contributions presented in the study are included in the article/**Supplementary Material**. Further inquiries can be directed to the corresponding authors.

## Ethics Statement

This is an observational, non-interventional database study, re-utilizing the data from the Global Burden of Diseases, Injuries, and Risk Factors 2019 study for the purpose of addressing a research question. The need for ethics approval and consent was waived.

## Author Contributions

FW and CW wrote this article and analysed the data. YL and HX prepared the figures. PY, SY and DZ designed the study and revised the article. All authors contributed to the article and approved the submitted version.

## Funding

The study was supported by Major Technological Innovation Special Project of Hubei Province of China (2019ACA167).

## Conflict of Interest

The authors declare that the research was conducted in the absence of any commercial or financial relationships that could be construed as a potential conflict of interest.

## Publisher’s Note

All claims expressed in this article are solely those of the authors and do not necessarily represent those of their affiliated organizations, or those of the publisher, the editors and the reviewers. Any product that may be evaluated in this article, or claim that may be made by its manufacturer, is not guaranteed or endorsed by the publisher.

## Abbreviations

95% CI, the 95% confidence interval; 95% UI, the 95% uncertainty interval; DALYs, disability-adjusted life-years; GBD, the Global Burden of Diseases, Injuries and Risk Factors Study; ICD, International Classification of Diseases; MI: mortality-to-incidence; YLDs, years lived with disability; YLLs, years of life lost
